# Current developments in delivering customized care: a scoping review

**DOI:** 10.1186/s12913-021-06576-0

**Published:** 2021-06-13

**Authors:** Etienne Minvielle, Aude Fourcade, Thomas Ricketts, Mathias Waelli

**Affiliations:** 1 i3-Centre de Recherche en Gestion, Institut Interdisciplinaire de l’Innovation (UMR 9217), École polytechnique, Batiment Ensta, 828, Boulevard des Maréchaux, 91762 Palaiseau Cedex, France; 2 Institut Gustave Roussy, 114, rue Edouard Vaillant, 94800 Villejuif, France; 3 University of North Carolina at Chapel Hill, Chapel Hill, North Carolina USA; 4 MOS (EA 7418), French School of Public Health, Rennes, France

**Keywords:** Personalized medicine, Mass customization, Organizational model, Patient-centered care, Care customization, Targeting population, Health care delivery, Logic of action

## Abstract

**Background:**

In recent years, there has been a growing interest in health care personalization and customization (i.e. personalized medicine and patient-centered care). While some positive impacts of these approaches have been reported, there has been a dearth of research on how these approaches are implemented and combined for health care delivery systems. The present study undertakes a scoping review of articles on customized care to describe which patient characteristics are used for segmenting care, and to identify the challenges face to implement customized intervention in routine care.

**Methods:**

Article searches were initially conducted in November 2018, and updated in January 2019 and March 2019, according to Prisma guidelines. Two investigators independently searched MEDLINE, PubMed, PsycINFO, Web of Science, Science Direct and JSTOR, The search was focused on articles that included “care customization”, “personalized service and health care”, individualized care” and “targeting population” in the title or abstract. Inclusion and exclusion criteria were defined. Disagreements on study selection and data extraction were resolved by consensus and discussion between two reviewers.

**Results:**

We identified 70 articles published between 2008 and 2019. Most of the articles (*n* = 43) were published from 2016 to 2019. Four categories of patient characteristics used for segmentation analysis emerged: clinical, psychosocial, service and costs. We observed these characteristics often coexisted with the most commonly described combinations, namely clinical, psychosocial and service. A small number of articles (*n* = 18) reported assessments on quality of care, experiences and costs. Finally, few articles (*n* = 6) formally defined a conceptual basis related to mass customization, whereas only half of articles used existing theories to guide their analysis or interpretation.

**Conclusions:**

There is no common theory based strategy for providing customized care. In response, we have highlighted three areas for researchers and managers to advance the customization in health care delivery systems: better define the content of the segmentation analysis and the intervention steps, demonstrate its added value, in particular its economic viability, and align the logics of action that underpin current efforts of customization. These steps would allow them to use customization to reduce costs and improve quality of care.

## Background

Increasingly, we encounter “customization”, ‘individualization”, and “personalization” terms in health care settings. Every patient wants to feel they are receiving optimized care, tailored to their particular needs, thus the traditional notion of a “doctor-patient relationship” implies customization is at the core of caregiving for health professionals [1]. Recently, customization has been combined with “personalized medicine”, which has evolved to “precision medicine” [2]. This takes the form of developed targeted therapies based on a patient’s genetic characteristics [3]. There is also a logical link between Patient-Centered Care (PCC) and care customization, where the care process considers individual patient preferences, needs and values [4, 5], often introducing personalization as a given component of the PCC package [6]. These notions have also been applied to individualized care plans, often managed by nurses [7], or to target care to the most vulnerable populations, or specific minorities, requiring a mix of clinical and social needs [8]. It is evident that many applications currently in practice or being proposed, fit under the “care customization” rubric.

However, it is unclear how these customized approaches are effectively delivered. These approaches converge on a care and service delivery model that could implement effective customized approaches into practices and organizations. These models, that are by definition, more flexible and demand-driven, could lead to better patient outcomes and experiences, and even if problematic, could reduce or avoid extra-costs [9]. But little is known about how these different approaches are effectively implemented and ultimately interacted in health care delivery systems. Some experiences remain experimental while others are disseminated without clear consensus on guidelines for implementing a customized effort in healthcare.

### The research question

Our research question is to understand how current personalization efforts that rely on multiple approaches are concretely implemented in healthcare delivery systems. For example, how are clinical or psycho-sociological patient characteristics selected and taken into accounts? And how are services and care established, assembled and combined for a specific patient? The answers to these questions can help to understand the benefits of care customization in practices and organizations. Aiming to address this gap in understanding, we conducted a scoping review in order to systematically map the literature on current efforts for delivering care customization. We selected this type of review rather than a systematic review because we addressed a broad topic where many approaches may be applicable. In order to determine how current approaches are actually implemented, it is also necessary to define the components of a customized delivery model and, therefore, to have an analytical framework. The “Mass Customization” (MC) concept is a benchmark in the field of customized delivery, illustrating a demand-driven model as opposed to a supply driven model [10].

### A conceptual framework for analyzing the literature

The concept of Mass Customization emphasizes the need to respond to the needs and preferences of the consumer or user, providing goods and services at or near mass production prices. Four elements must converge to generate an effective customized delivery [11–13]: (i) Design-based on a segmentation analysis of the user, often referred to as “customer relationship management”, where the goal is to precisely match demand to the service or product [14, 15]; (ii) Fabrication, where the system (factory, service unit or team) adapts production to meet each of those needs; (iii) Assembly, where sub-systems or “modular” service structures flexibly combine multiple standardized products and services [11, 16]; and (iv) Distribution, where coordination and integration of different products and services facilitate timely delivery to the customer [17, 18]. “Personalization” is also used in this context, and describes the employees’ attitudes, so a customer’s preference is catered for [19]. These four stages of MC define two phases of any customization delivery approach: (i) segmentation analysis of individual user characteristics, and (ii) customized intervention (i.e. fabrication, assembly and distribution), and modularity related issues [20].

The transfer of MC to the health sector must be considered carefully, because it comes up against several limitations. The objectives pursued in commercial and public health sectors are quite different. Similarly, consumer and patient needs differ accordingly. In respect of these limits, several researches have studied how to apply MC in the health care field. In their early and seminal paper on customization, Lampel and Minzberg [21] provided multiple examples related to care, stressing how much the patient’s clinical condition already lent itself to personalization (i.e. cataract surgery in a healthy person is much less customizable than a complex cardiac surgery, thus it proceeds differently). Recently, Mannion and Exworthy [22] reactivated a debate on the antagonism between standardization and customization, making service delivery homogeneous, but anonymous and customizable [23], thereby requiring different combinations of customization/standardization in care activities. Minvielle et al. [24] underscored the conditions for implementing a MC model along the care pathway. Several studies also proposed care services in a modular format, using a menu of options adapted to individual patients or unit needs, in different care contexts [25–29]. All these approaches converge to demonstrate how to implement MC in health care. In contrast, there are no studies that apply the concept of MC to assess current efforts to develop customized care.

The use of this concept seemed relevant to us within the framework of our research because even if the objectives are not the same, the steps of delivery of a customized service are identical from one sector to another. Thus, this conceptual framework has helped to identify how these developments address the two key-components of segmentation analysis and customized intervention. Likewise, it has also made it possible to document the characteristics of the patients for whom these approaches were used, the goals for which they were designed as well as the evidence of their added value, and the very type of customized interventions carried out.

## Methods

This review followed Preferred Reporting Items for Systematic Reviews and Meta-Analyses (PRISMA) guidelines for scoping reviews [30], according to the five steps of such a review methodology: (i) identifying the research question (describe in the previous section), (ii) identifying relevant studies, (iii) study selection, (iv) charting the data, and (v) collating, summarizing and reporting the results [31, 32].

To identify potentially relevant documents (step 2), the following bibliographic databases were searched from January 2008 to March 2019: MEDLINE, Scopus, JSTOR, Web of Science, Pubmed, Psycinfo and Science Direct. This period was selected as it reflected recent/current efforts in delivering customized care. Searches were limited to English-language articles. The search was focused on articles that included “care customization”, “personalized service and health care”, individualized care” and “targeting population” in the title or abstract. These words as well as the following have been selected because they are conventionally used in the healthcare sector in the name of care customization. Following the principle of “narrowing” the topic [31, 33], we instigated a second Boolean search strategy on Web of Science using the following keywords: (1) care customization OR personalized medicine OR personalized service OR targeting populations OR individualized care AND (2) segmentation analysis OR profiling patients OR categorizing patients AND (3) patient needs OR patient characteristics OR patient preferences OR population characteristics OR patient demands (see appendix 1 for an example of an entire search strategy). In total, 6390 articles were identified. The search results were exported into Mendeley, and duplicates were removed by a reviewer (AF) at each iteration.

Inclusion and exclusion criteria were defined to identify the final sample of articles (step 3). We included any studies addressing aspects of segmentation analysis and customized intervention, in real health care delivery contexts. We defined “segmentation analysis” as any analysis of patient characteristics, even without explicit reference to specific customization methods, but with the objective of customizing delivered care and services. We also defined “customized interventions” as any developments in health supply that implemented targeted answers, no matter how the care team/process was designed, assembled or delivered, and regardless of the type of practice or organization (i.e. individual, small or large group). Lastly, we did not predefine how segmentation analysis and interventions interacted, and we accepted critical analyses and reviews of care customization strategies when they addressed both key parts of segmentation analysis and customized intervention, as we defined it. We tested our selection criteria on 20 articles (randomly extracted from the first literature review) to determine criteria modification. Accordingly, a second inclusion and exclusion criteria list was defined to refine our final selection.

### Inclusion criteria

1. Full-text in English.
2. The notion of customization and/or targeting and/or individualized and/or personalization in association with health care.
3. Patient needs, demands or preferences were addressed to create a customized strategy, related to a segmentation analysis in a health care delivery context.
4. The development or design implementation, sustainability, and/or dissemination of a customized intervention using a segmentation analysis. Articles were included if they explicitly reported a customization approach (i.e., population mapping, assessment of tailored interventions and properties, or analysis of a MC approach).

### Exclusion criteria:

1. Care customization was mentioned, but segmentation analysis and/or customized interventions were not reported (i.e. the primary focus was human or technological support, or other related topics on care customization, without documenting a segmentation analysis and customized intervention; the customization approach was only expressed as a recommendation or a requirement).
2. Segmentation analysis was not followed by a customized intervention in the care delivery system (i.e. genomic studies that suggested new targeted therapies, or questionnaires assessing patient quality of life; segmentation analyses from a technical or methodological viewpoint (i.e. selection bias, regression analyses), or analysis of patient needs, without reporting a customized intervention).
3. Segmentation analysis and/or customized intervention as a strategy for a clinical trial, fundamental research purposes or reviews, articles not based in the real world of care delivery (i.e. randomized trials assessing a real-world intervention were included).
4. Articles investigating a customized intervention without referring to a segmentation analysis.
5. Any stratification method or regression analysis unrelated to a customized intervention (i.e. analyzing relationships between a set of factors (i.e. patient behaviors), and an outcome; or customized clinical scores predicting outcomes, without an explicit customized strategy of care).
6. The customization approach is applied outside health care, or to any health care delivery aspect, but not to patient characteristics (i.e. electronic health records or monitoring tools).
7. Articles comprising comments, commentaries, dissertations or conference proceedings.
8. To increase consistency among reviewers, two investigators screened the same 200 first abstracts (AF, EM). Titles and abstracts were then independently reviewed by one investigator (EM). Abstract analyses generated 240 full-text articles which were independently assessed for review eligibility by the two same reviewers. Consensus was agreed for uncertain cases through discussion between the two reviewers, and in three cases with a third reviewer (MW). Last, the analysis was supplemented by the manual review of eligible bibliographies for inclusion (six articles were added).

The last steps 4 (charting the data) and 5 (collating, summarizing and reporting the results) led to coding the data contained in the articles according to the components of the concept of Mass Customization. This resulted in the definition of three broad categories (the first and third being descriptive while the second refers to the MC’s framework): 1) context characteristics (publication year; sample country/location; health topic; type of review); 2) customized strategy (applied versus potential/proposed interventions; patient characteristics and type of patient characteristics supporting the segmentation analysis; intervention; impact evaluation), and 3) use of a conceptual basis/theory (articles that mentioned MC; other theories informing a conceptual basis related to care customization because we hypothesized that in this exploratory field other references could be mobilized) (see Table 1 in the result section). For each article, the methodological characteristics, topics and the customization process including the two components of segmentation analysis and customized intervention of the concept of MC were also described (see Table 2 in the result section). Finally, we did not analyze methodological qualities in our studies, which were heterogeneous. However, our goal was not to uncover evidence on specific customized delivery models, but to qualitatively extract information from the literature.

**Table 1:** Summary of study articles

Characteristics	Content
**MATERIAL**	
**Publication Year: No of articles**	2008–2011: 5 2012–2015: 23 2016–2019: 42
**Study country/location**	Australia: 6, Canada: 4, China: 1, Denmark: 1, Finland: 2, France: 1, Germany: 2, India: 1, Indonesia: 1, Iran: 1, Israel: 1, Italy: 2, International consortium: 5, Japan: 1, The Netherlands: 7, Philippines: 1, Switzerland: 1, Taiwan: 1, United Kingdom: 4, USA: 29
**Health Topic**	Acute orthopedic surgery: 1 Cancer: 13 Thyroid cancer: 1 Breast cancer: 2 Radiotherapy: 1 Cancer screening: 3 Lung cancer: 1 Cancer: 1 Head and neck cancer: 1 Cancer survivors: 1 Urological cancer: 1 Cancer-related fatigue of patients with ovarian cancer: 1 Children with autism spectrum disorders: 1 Children with attention-deficit/hyperactivity disorder (ADHD): 1 Children with learning disabilities: 1 Children with obesity: 1 Chronic conditions: 2 Chronic disease (prevention): 2 Dementia/mental illness: 3 Diabetes 2: 1 Diversity: 1 Elderly: 9 End-of-life: 1 Genomic characteristics: 2 Hemophilia: 1 High needs high costs: 3 Higher utilizers: 5 Homeless: 1 Hospitalized patients: 1 Immigrants: 1 Kidney transplantation: 1 Lesbian, Gay, Bisexual and Transgender (LGBT): 1 Marginalizing conditions: 1 Medical tourism: 1 Obesity: 1 Pediatric palliative care: 1 Pregnant women: 1 Sickle cell disease: 1 Spinal cord injury: 1 Transversal (all patients): 8 Visual impairment: 1 Women friendly-environment: 1
**Type of review**	Administrative report: 2 Engineering: 1 Health service research: 23 Medical: 28 Nursing: 9 Pharmaceutical: 2 Social science: 5
Care Customization Efforts	
**Applied versus potential/proposed intervention**	Applied: 27
	Potential/proposed: 43
**Segmentation Analysis**	
***Patient characteristics***^1^	
**Type of patient characteristics associated**	Clinical: 51
	Cost: 8
	Psychosocial: 39
	Service: 30
	All types, no clear definition: 11^2^
	No association:
	Clinical needs: 12
	Service needs: 1
	Psychosococial needs: 2
	One association:
	Clinical and psychosocial needs: 11
	Clinical and service needs: 1
	Clinical needs and cost: 3
	Psychosocial and service needs: 4
	Two associations:
	Clinical, psychosocial and service needs: 19
	Three associations:
	Clinical, psychosocial, service needs and cost: 5
**Intervention**	No precision: 1
	Fabrication: 20
	Assembly: 7
	Distribution: 54
**Impact evaluation**^**3**^	No: 52
	Yes: 18
	Cost: 6
	Negative personality traits associated with expensive health care services: 1
	Reducing health care costs for high needs high costs: 1
	Reducing readmissions: 2
	Reducing Emergency Department (ED) and length of stay for high utilizers: 2
	Experience: 7
	Improving care provision: 1
	Communication: 2
	Patient experience: 1
	Patient experience (homeless): 1
	Quality-of-life: 2
	Quality: 5
	Appropriate treatment: 1
	Depressive symptoms: 1
	Monitoring LDL (Low Density Lipoprotein*)* cholesterol: 1
	Weight reduction: 1
	Better outcomes: 1
Theories	
**Articles referring to MC**	Yes: 6
	No: 64
**Theories informing conceptual basis related to Care Customization (*n*** **= 20)**	Collaborative intervention planning framework: 1
	Comprehensive geriatric assessment: 1
	Consumer-direct care: 1
	Equity-oriented health care model: 1
	Essential care model: 1
	Individualized care: 6
	Inter-organizational collaboration: 1
	Modularity service: 1
	Optimization of personalized assortments: 1
	Organizational approach of diversity: 1
	Patient-centered care: 3
	Patient-centered segmentation: 2
	Patient needs: 1
	Patient profiling: 1
	Personality factors and traits: 3
	Personalized and precision medicine: 5
	Population strategies in integrated care: 1
	Professional practice environnement: 1
	Psychosocial marketing segmentation technique: 1
	Quality assurance method: 1
	Social marketing theories (social learning theory, health belief model): 1
	Trans theoretical Model: 1

^1^Articles could develop more than one of the four types of patient characteristics, and more than one of the three intervention steps, as well as more than one theory as a conceptual basis. For more details, see Table 2

^2^In some cases, segmentation analysis was mentioned, but without any explicit definition of patient characteristics

^3^The impact evaluation list is in Table 2

**Table 2:** Description of the methodological characteristics, topics and the customization process (*n* = 70)

Author (s) & year of publication	Population & methodology	Health topics and objectives	Customization implications (Segmentation method: patient characteristics & types of method; intervention content: fabrication, assembly, distribution) MC and other conceptual basis	Evidence of added-value
1. Aggarwal et al. (2018)	**USA**, Quantitative study (Questionnaire analysis of 644 patients in Mwanza, Tanzania at three clinics)	Visual impairment in developing countries, Identifies individualized needs in local communities in two African countries	**Segmentation analysis:** Clinical and psychosocial needs (e.g. the perceived expense and lack of vision problems) **Intervention:** Targeting the needs of local communities by supplying optometric or ophthalmic services (distribution)	
2. Avis et al. (2013)	**Canada**, Quantitative study (Comparative analysis of a pre-post intervention of 165 children with obesity)	Children with obesity, Demonstrates the added-value of an individualized plan	**Segmentation analysis:** Clinical, psychosocial and service needs (e.g. anthropometric, li*f*estyle, medical and behavioral attitudes) **Intervention:** A multidisciplinary team managing an individualized plan (counseling and education related to nutrition and behavioral change techniques) (distribution)	**Quality of care** Weight stabilization and a modest weight reduction
3. Barlow-Stewart (2017)	**Australia**, Recommendations (Literature Review)	Genomic information, Highlights personalized medicine challenges in healthcare delivery	**Segmentation analysis:** Clinical needs (genomic characteristics) **Intervention:** A customized plan including consent forms, data storage and analysis of genomic information; appropriate genomic literacy and genetic counselors) (fabrication and distribution) **Conceptual basis:** Precision medicine/personalized medicine	
4. Blanch-Hartigan and Viswanath (2015)	**USA** Quantitative study (Regression analysis of 519 cancer survivors)	Cancer Customizes cancer-related information according to demographical status	**Segmentation analysis:** Clinical and psychosocial needs (i.e. social determinants predicting differences in cancer-related information) **Intervention:** Target communication platforms based on demographic profiles of survivor audiences (e.g. African American cancer survivors) (distribution)	
5. de Blok et al. (2010)	**The Netherlands** Qualitative study (four case studies)	Elderly, Demonstrates the impact of modularity packages on care customization in independent elderly people	**Segmentation analysis:** Clinical and service needs (e.g. housing, support services) **Intervention:** A package of care and services for the elderly (built around key components of homecare activities, social activities, and an alarm service. Adapted to patient needs (fabrication, assembly, distribution) **Conceptual basis:** Modularity service and MC	
6. Blumenthal et al. (2016)	**USA** Recommendations (case study review)	High-needs, high-costs Tailors complex care management, coordination, and integration for high-needs, high-cost patients	**Segmentation analysis:** Clinical, psychosocial, service needs and costs **Intervention:** Complex care management program (including population segmentation, non-medical services, care manager, interdisciplinary teamwork) (fabrication, distribution)	**Cost** Reduction of hospital use and costs
7. Bos-Touwen et al. (2015)	**The Netherlands** Qualitative study (Semi-structured interviews with 15 nurses in chronic care, in different settings)	Chronic patients Explores how nurses assess chronic patients and investigates the potential for self-management and clinical reasoning with regard to tailoring care	**Segmentation analysis:** Psychosocial needs (self-management attitude, i.e. four patient types: unmotivated patients, patients with limited capacities, oblivious patients and ideal patients) **Intervention:** Different approaches depending on the nurse’s perception of patient self-management (distribution)	
8. Bosua et al. (2016)	**Australia** Recommendation (literature review)	Mental illness/elderly Develops an integrated information technology (IT) framework that supports customized treatment plans for adults with mental illness in residential care facilities	**Segmentation analysis:** All needs, but no clear definition (e.g. information needs for individualized mental illness treatment plans) **Intervention:** An innovative IT solution (i.e. a portal with centralized information storage) (distribution)	
9. Braaf et al. (2017)	**Australia** Qualitative study (22 in-depth interviews)	Patients living with spinal cord injury Explores the needs of people with spinal cord injury, receiving formal carer and hospital services	**Segmentation analysis:** Clinical, psychosocial and services needs **Intervention:** More reliable and accessible supply of carers. Individualized care plan in hospital, rehabilitation, and community settings (distribution)	
10. Brandzel et al. (2017)	**USA** Qualitative study (Adult focus groups recommended for cancer screening)	Cancer screening Customizes cancer screening reminder’s messages	**Segmentation analysis:** Psychosocial and service needs (e.g. age, preference and health beliefs) **Intervention:** Customized forms and timing of reminders (e.g. electronic, paper, telephone) (distribution)	
11. Cabassa et al. (2014)	**USA** Qualitative study (case study)	Hispanic patients with serious mental illness Customizes an existing healthcare manager intervention, for a new patient population (Hispanics).	**Segmentation analysis:** Clinical, psychosocial and service needs (e.g. patient-provider relationship) **Intervention:** Provider level-adaptations without compromising the core elements of a healthcare manager intervention (distribution) **Conceptual basis:** Collaborative intervention planning framework	
12. Chaudhuri and Lillrank (2013)	**India and Finland** Qualitative study (Literature review and a case study)	Transversal Implements MC in the Indian healthcare system	**Segmentation analysis:** All needs, but no clear definition **Intervention:** Six dimensions (demand management, supply management, service process design, quality management, job design and resource profiling, scheduling) Application of the MC intervention requires a high volumes of patients enabling sub-specialization with sufficient capacity utilization **Conceptual basis:** MC	
13. Cotrell & Carder (2010)	**USA** Qualitative study (interviews with 130 residents)	Elderly Identifies resident unmet needs to target services	**Segmentation analysis:** Clinical and psychosocial needs **Intervention:** Appropriate skills for managers and service coordinators to assess bio-psycho-social functioning of older residents (e.g. ethnic or language) (distribution)	
14. Von Dadelszen et al. (2015)	**International** Recommendations (a consensus based on an international initiative)	Pregnant women Provides evidence-based personalized care to women, wherever they encounter the health system	**Segmentation analysis:** Clinical needs (i.e. risk-stratification) **Intervention:** Integrated approach to identify women, fetuses, and newborns requiring facility-based care and to initiate lifesaving interventions prior to transportation (distribution)	
15. Dekkers and Hertroijs (2018)	**The Netherlands** Qualitative study (comparative analysis of two case studies)	Transversal Customizes care based on two profiling techniques for patients	**Segmentation analysis:** Clinical, psychosocial and service needs **Intervention:** Customized care depending on a specific profiling technique (distribution) **Conceptual basis:** Patient profiling and MC	
16. Dewi et al. (2014)	**Indonesia** Qualitative study (Case study based on professionals experiences)	Transversal Implements an individualized care plan in the Indonesian health-care system	**Segmentation analysis:** All types, but no clear definition **Intervention:** Patient-nurse assortments based on similarity in social status (distribution) Responsiveness to local conditions by encouraging decision-making capacity, and developing skills, abilities and motivation of local officials working in the health sector (distribution) **Conceptual basis:** Patient-centered care	
17. dosReis et al. (2017)	**USA** Quantitative study (Conditional logit model method; 184 primary caregivers)	Childhood attention-deficit/hyperactivity disorder (ADHD) Identifies ADHD management options that caregivers prefer	**Segmentation analysis:** Service needs (i.e. caregiver preferences) **Intervention:** Assessing preferences over the course of care to facilitate patient/family-centered care planning across a range of resources, and a multidisciplinary team (fabrication, assembly, distribution)	
18. Fertel et al. (2019)	**USA** Quantitative study (Comparative analysis of a pre-post intervention with a control group and involving 452 patients)	High utilizers Assesses whether individualized care plans (ICPs) reduce costs, inpatient length of stay, and Emergency Department (ED) encounters in a large healthcare system	**Segmentation analysis:** Clinical needs and costs (i.e. high utilizers defined by the number of ED visits per year) **Intervention:** An individualized care plan including specific symptom related information;assessments by specialists;social work and psychiatry summary (distribution)	**Costs** Reduce costs, inpatient length of stay and ED visits for high utilizers
19. Flott et al. (2017)	**United Kingdom** Quantitative study (Two-step cluster analysis of 17,520 urological cancer patients)	Urological cancer Improves patient experiences by defining priority groups	**Segmentation analysis:** Clinical, psychosocial, and service needs (e.g. gender, age, cancer type and income level) **Intervention:** Access to a general practitioner when required for specific groups (e.g. marginalized social groups) (distribution)	
20. Ford-Gilboe et al. (2018)	**USA** Quantitative study (Path analysis method involving 395 patients)	Patients living in marginalized conditions Demonstrates the impact of an equity-oriented health care intervention	**Segmentation analysis:** Clinical, psychosocial (e.g. poverty) and service needs (e.g. scheduled appointment) **Intervention:** A set of strategies applied by primary care professionals (e.g. routinely inquire about access to health determinants, such as food, shelter, clothing, and the impact of financial strain) (distribution) **Conceptual basis:** Equity-oriented health care model	**Quality of care & patient experience** Greater patient comfort and confidence improves health outcomes (i.e. depressive symptoms, chronic pain, quality of life)
21. Friedman et al. (2013)	**USA** Quantitative study (Regression analysis of 1605 participants)	Higher users Tests the hypothesis that a negative personality trait (neuroticism) is associated with greater health care use; ED visits, and nursing home use	**Segmentation analysis:** Clinical and psychosocial needs **Intervention:** Considering personality traits in customized intervention (i.e. five personality traits: neuroticism, extraversion, openness to experience, agreeableness, and conscientiousness) (distribution) **Conceptual basis:** Personality traits	**Cost** Personality traits (neuroticism) are associated with expensive health care services
22. Geller et al. (2008)	**USA** Quantitative study (Mixed model analysis of 319 patients)	Colorectal screening Assesses the impact of a computer tablet, patient/provider, communications assistant	**Segmentation analysis**: Clinical and psychosocial needs **Intervention:** Personalized information (i.e. to use language, concepts, and visuals for people with varying degrees of education and health literacy) rather than tailored interventions based on literacy levels (distribution) **Conceptual basis:** Trans theoretical model	
23. Gesser-Edelsburg & Shalayeva (2017)	**Israel** Qualitative study (18 interviews and 80 comments from nutrition forums users)	Nutrition program for immigrants Investigates factors influencing the success of nutrition programs. Internet-based	**Segmentation analysis:** Psychosocial and service needs (e.g. immigrant cultural characteristics and patterns of internet use) **Intervention:** Customized information depending on language and cultural habits (distribution)	
24. Golden et al. (2019)	**USA** Recommendations (literature review)	Elderly Implements a care model that customizes care according to the needs of older adults with serious illness, and their families	**Segmentation analysis:** Clinical, psychosocial and service needs **Intervention:** Racially and ethnically diverse healthcare professionals, including mental health and direct service workers (distribution) An integrated network of community-based organizations providing in-home services (fabrication and assembly) An electronic communications platform that spans providers and organizations with skilled technology staff (distribution) **Conceptual basis:** The essential care model	
25. Gordon et al. (2014)	**USA** Quantitative study (Cluster analysis based on a marketing segmentation technique in 102 African American clinic patients)	Colorectal cancer screening To customize messages for colorectal cancer screening orientation among African American clinic patients with limited literacy	**Segmentation analysis:** Clinical, psychosocial and service needs (e.g. preventive health-related attitudes, values, preferences and behaviors) **Intervention:** Tailoring health messages and improving medical communication based on their preventive health perceptions (distribution)	
26. Greenwalt et al. (2020)	**USA** Recommendations (literature review)	Breast cancer Develops precision medicine for breast cancer management	**Segmentation analysis:** Clinical needs (i.e. precision medicine criteria including molecular subtyping and gene expression profiles) **Intervention:** Customized treatments based on multi-gene assays, molecular and expression profiling (fabrication) **Conceptual basis:** Precision medicine/ personalized medicine	
27. Hagan et al. (2017)	**USA** Qualitative study (Content analysis of 47 patients)	Cancer-related fatigue (CRF) in patients with ovarian cancer Describes cancer patient goals and strategies for managing CRF	**Segmentation analysis:** Clinical and psychosocial needs (e.g. enjoying time with friends and family, having energy to be physically active) **Intervention:** 11 customized strategies according to the specific needs of a patient (e.g. dealing with emotions; energy conservation, etc.) (distribution)	
28. Hardin et al. (2017)	**USA** Qualitative study (Case study in two settings)	High needs high costs Assesses the impact of an inter-organizational collaboration for complex patients	**Segmentation analysis:** Clinical, psychosocial, service needs and costs (i.e. high-frequency patients that overuse acute care services) **Intervention:** Inter-organizational infrastructure and practices facilitating effective cross collaboration between competing health systems (e.g. shared infrastructure, common individualized plans between hospital and inpatient team) (assembly and distribution) **Conceptual basis:** Inter-organizational collaboration	**Cost** Reduction in healthcare utilization and costs for this population
29. Hassett et al. (2012)	**USA** Quantitative study (Regression analysis based on a prospective registry data set)	Breast cancer Assesses the impact of a genomic test to customize chemotherapy	**Segmentation analysis:** Clinical needs (i.e. genotype profile) **Intervention:** A gene expression profile test for the appropriating use of adjuvant chemotherapy (fabrication) **Conceptual basis:** Precision medicine/personalized medicine	**Quality of care** Reduction in the proportion of women with a specific genomic expression profile, receiving adjuvant chemotherapy
30. Hooper et al. (2013)	**USA** Quantitative study (Comparative analysis of a pre-post intervention in 78 patients in a health care center)	Kidney transplantation Develops and evaluates a system for individualized risk-based monitoring of cholesterol and 11 other tests, after kidney transplantation in children	**Segmentation analysis:** Clinical needs (i.e. dyslipidemia risk) **Intervention:** Monitoring schedules individualized by dyslipidemia risk Automated pre-visit decision support from electronic medical records Automated results forwarding to providers (fabrication) **Conceptual basis:** Quality assurance methods	**Quality of care** Significant improvements in the numbers of patients with controlled LDL cholesterol
31. Hunter et al. (2016)	**Canada** Quantitative study (Regression analysis of 109 long-term care staff)	Dementia Assesses associations of personal, organizational and environmental characteristics with self-reported person-centered behaviors in long-term residential care settings	**Segmentation analysis:** Not clear definition, all types **Intervention:** Organizational and individual characteristics of staff members (including gender, beliefs on personhood and burnout) for improving person-centered dementia care (e.g. respect for personhood and comfort care) (distribution) **Conceptual basis:** Patient-centered care	
32. Jackson et al. (2019)	**International** Qualitative study (case reports)	People with hemophilia Personalizes prophylaxis in hemophilia	**Segmentation analysis:** Clinical, psychosocial and service needs (e.g. desire for strong physical activity) **Intervention:** Customized treatment according to patient lifestyles (distribution)	
33. Jenq et al. (2016)	**USA** Quantitative study (difference-in-difference analysis in 10,621 discharge patients aged > 64 years, with Medicare insurance)	Higher utilizers Evaluates whether Medicare FFS readmissions were reduced via a customized intervention applied to high-risk discharge patients	**Segmentation analysis:** Clinical needs and costs **Intervention:** A personalized transitional care plan managed by transitional care consultants, involving education, medication reconciliation, follow-up telephone calls, and links to community resources (distribution)	**Cost** Reduce readmissions in the population (despite being only delivered to high-risk patients)
34. Jing et al. (2012)	**International** Qualitative study (case reports)	Transversal Customizes molecular genetic data and health knowledge into a standard-based electronic health record (EHR) prototype	**Segmentation analysis:** Clinical needs **Intervention:** Customized genetic and clinical characteristics via a standard-based EHR system (fabrication & distribution) **Conceptual basis:** Precision medicine/ personalized medicine	
35. Kertesz et al. (2013)	**USA** Quantitative study (Comparative analysis of 634 participants)	Homeless Assesses the impact of tailored primary care programs in homeless patients	**Segmentation analysis:** All needs, but no clear definition **Intervention:** Tailored primary care program based on four dimensions (patient-clinician relationship, perception of cooperation, access/coordination and consideration of homeless-specific needs) (distribution)	**Patient experience** A better service experience for patients who experience homelessness
36. Kolodinsky and Reynolds (2009)	**USA** Quantitative study (Cluster analysis of 581 citizens)	Obesity Identifies elements of the US overweight population for message targeting	**Segmentation analysis:** Clinical, psychosocial and service needs (e.g. overweight status, activity levels, health and food behaviors) **Intervention:** Customized channels and messages according to consumer lifestyles and their needs (distribution) **Conceptual basis:** Social marketing theories (social learning theory, health belief model)	
37. Kusch et al. (2018)	**Germany** Recommendations (literature review)	Transversal Customizes drug information according to individual information needs	**Segmentation analysis:** Clinical needs (i.e. preferences about drug information on adverse drug reactions and drug-drug interactions) **Intervention:** Customized drug information according to individual information needs (distribution) **Conceptual basis:** Patient-centered care	
38. Van der Laan et al. (2014)	**The Netherlands** Quantitative study (Factor mixture model in 2019 older adults)	Elderly A new care delivery system based on a person-centered segmentation, beyond clinical needs	**Segmentation analysis:** Clinical and psychosocial needs (e.g. restrictions in coping; mobility needs; feeling alone; cognitive needs) **Intervention:** A set of care and service modules depending on patient needs, related to five segments (fabrication and assembly) **Conceptual basis:** Patient needs; patient-centered segmentation	
39. Larsen et al. (2019)	**Denmark** Quantitative study (including a regression analysis of 9400 patients)	Prevention of chronic disease Implements an intervention (a personal digital health profile, followed by a targeted preventive program for high-risk patients)	**Segmentation analysis:** Clinical and psychosocial needs (i.e. clinical risk and health risk behaviors) **Intervention:** A personal digital health profile for recruiting patients to preventive programs across primary care providers (fabrication)	
40. Lattie et al. (2016)	**USA** Quantitative study (Cluster analysis of 212 men with a prostate cancer diagnosis)	End-of-life care Examines whether the five-factor personality model explains variations in preferences for end-of-life intervention in men with prostate cancer	**Segmentation analysis:** Clinical and psychosocial needs **Intervention:** Customized interventions according to three preferences related-groups, Comfort-Oriented Patients (preferring palliative care and opposing life support services), Service-Accepting Patients (preferring both palliative care and life support), and Service-Reluctant Patients (preferring neither) that endorsed significantly higher levels of neuroticism (emotional instability and negativity) (distribution) **Conceptual basis:** Personality traits	
41. Leporatti et al. (2016)	**Italy** Quantitative study (Regression analysis of 218,198 ED visits)	Emergency Department (ED) users Investigates the characteristics of frequent ED users, and recommends alternative medical services for such patients	**Segmentation analysis:** Clinical, psychosocial, service needs and costs (e.g. abuse of alcohol and drugs, chronic conditions, and psychological distress) **Intervention:** To extend primary care services outside ED (distribution) and towards instituting local aid services (fabrication)	
42. Lin (2011)	**Taiwan** Quantitative study (Questionnaire survey with 154 replies)	Female-friendly hospital environment Analyzes womens’ traits and needs to determine female-friendly hospital environments	**Segmentation analysis:** Clinical, psychosocial and service needs **Intervention:** Female-friendly hospital environments (e.g. women’s consultation or ladies’ rooms (fabrication); verbal sensitivity of gender awareness (distribution)	
43. Loeffen et al. (2018)	**The Netherlands** Qualitative study (case study)	Pediatric palliative care Implements a customized intervention for advanced care planning and anticipatory actions	**Segmentation analysis:** Clinical, psychosocial and service needs (e.g. end-of-life care; links with school) **Intervention:** An individualized pediatric palliative care plan built on five domains (data, basics, social, psychosocial and spiritual and physical care), and promoting collaboration and anticipatory planning and action (distribution). **Conceptual basis:** individualized care	
44. Manegold (2014)	**Germany** Recommendations (literature review)	Advanced non-small cell lung cancer Customizes therapy for histo-typing and genotyping tumors	**Segmentation analysis:** Clinical needs **Intervention:** Routine molecular testing for health care delivery (fabrication)	
45. McCabe et al. (2019)	**Australia** Quantitative study (Comparative analysis of 92 residents)	Elderly Assesses the impact of a customized consumer direct care model on resident quality of life	**Segmentation analysis:** All types, but no clear definition **Intervention:** Development of staff skills in communicating with residents, organizational change and transformational leadership (distribution) **Conceptual basis:** Consumer-direct care	**Patient experience** Significant improvement in resident quality of life
46. McConnell et al. (2017)	**United Kingdom** Quantitative study (Descriptive analysis based on data from UK registries)	Cancer patients Categorizes patients (clusters of common treatment aims, experiences and outcomes) to provide a numerical framework of services according to the needs of people with different cancers	**Segmentation analysis:** Clinical needs **Intervention:** To include data on treatment regimens, patient preferences, needs, attitudes and behaviors in group descriptions, as this information is not routinely collected in cancer registration data. To deliver personalized care based on a high-level view of potential care requirements to support service planning (fabrication and distribution)	
47. Mercer et al. (2015)	**USA** Quantitative study (Comparative analysis of a pre/post intervention in 24 medically and psychosocially complex patients, with the highest rates of inpatient admissions and ED visits during a one year-analysis)	High utilizers Develops individualized care plans to reduce unnecessary healthcare service utilization and hospital costs for complex, high utilizers of inpatient and ED care	**Segmentation analysis:** Clinical, psychosocial and service needs and costs (i.e. having at least three ED visits or admissions within six months and some degree of medical, social, or behavioral complexity) **Intervention:** A multidisciplinary team and individualized care plan (distribution)	**Cost** Significantly reduce hospital admissions (after six months), 30-day readmissions and hospital costs for complex, high-utilizers
48. Minvielle et al. (2014)	**France** Recommendations (literature review)	Transversal Develops a new customized care delivery model	**Segmentation analysis:** Clinical, psychosocial and services needs **Intervention:** A customized delivery model combining categorization systems, information technology, patient engagement and nurse navigators (distribution) **Conceptual basis:** MC	
49. O’Malley et al. (2019)	**USA** Qualitative study (Semi-structured interviews of 34 leaders from mature Accountable Care Organizations (ACOs) and national experts	High needs high costs (HNHC) Explores how a group of mature ACOs match patients with appropriate interventions, by segmenting HNHC populations with similar needs, into smaller subgroups	**Segmentation analysis:** Clinical, psychosocial, service needs and costs **Intervention:** Segmentation analysis and an understanding of what skill sets and staff are needed to deliver enhanced care management (distribution)	
50. Oulton et al. (2015)	**United Kingdom** Qualitative study (Ethnographic study in 27 hospital staff)	Children with learning disabilities Understanding the needs (individualized care) of children and young people with learning disabilities, and their families during hospitalization.	**Segmentation analysis:** Clinical, psychosocial and service needs **Intervention:** Focusing on the “little things” (e.g. music therapy) Creating a safe and familiar environment, accessing and using appropriate equipment, developing partnerships with parents (distribution)	
51. van Overveld et al. (2018)	**The Netherlands** Qualitative study (Interviews with 14 patients and chairpersons of two patients associations)	Head and neck cancer, Incorporating patient needs and preferences in integrated care	**Segmentation analysis:** Psychosocial and service needs **Intervention:** Personalized communications, education and information that meets patient requirements. Adequate involvement of allied health professionals for physical support, and family and friends in aftercare (distribution) **Conceptual basis:** Patient-centered segmentation	
52. Pan et al. (2019)	**China** Qualitative study (case study)	Transversal Delivering personalized recommendations of physician assortments to patients with heterogeneous illnesses, and selecting one physician according to patient preferences	**Segmentation analysis:** Clinical and psychosocial needs (i.e. preferences about physician profiles) **Intervention:** An algorithm supporting a web-based appointment system that optimizes physician-patient assortments according to patient preferences (fabrication) **Conceptual basis:** Customized dynamic assortment planning with demand learning	
53. Papastavrou et al. (2015)	**International** Quantitative study (Survey questionnaire of 1163 nurse’s perception across seven countries)	Acute orthopedic and trauma surgical inpatients Assessing if nurses’ views of their professional practice environments are associated with their views on care individualization levels	**Segmentation analysis:** All types, but no clear definition **Intervention:** Considers professional practice environment elements associated with care individualization (e.g. internal work motivation, cultural sensitivity, teamwork and staff relationships with physicians) (distribution) **Conceptual basis**: The revised professional practice environment model; individualized care	
54. Peter & Lupsa (2019)	**USA** Recommendations (literature review)	Diabetes type 2 Personalizing the management of patients with type 2 diabetes	**Segmentation analysis:** Clinical, psychosocial and service needs **Intervention:** Tailoring medical therapy (distribution)	
55. Petry et al. (2019)	**Switzerland** Qualitative study (Semi-structured, individual or dyadic interviews with 19 elderly patients)	Elderly patients with cognitive impairments Understanding the experiences and needs of older persons with cognitive impairment, and their families	**Segmentation analysis:** Clinical, psychosocial and service needs (with a focus on family members) **Intervention:** A customized intervention based on various dimensions (e.g. caring attentiveness and responsiveness, access to staff and information, participation in care, and support over time) (distribution)	
56. Pilotto et al. (2017)	**Italy** Recommendations (literature review)	Elderly Assessing the impact of a Comprehensive Geriatric Assessment (CGA) model in determining clinical profiles, pathological risks and prognoses, and facilitating clinical decision-making on the personalized care plans of older persons	**Segmentation analysis:** Clinical and psychosocial needs (e.g. age, medical comorbidities, psychosocial problems, previous or predicted high healthcare utilization, changes in living situation, and specific geriatric conditions) **Intervention:** Tailored programs in older frail patients based on CGA programs **Conceptual basis:** A comprehensive geriatric assessment	
57. Powell et al. (2018)	**USA** Quantitative study (Comparative analysis of a pre-post intervention in 242 sickle cell disease (SCD) patients)	Sickle cell disease patients Assessing the impact of a customized intervention on ED visits and hospitalization	**Segmentation analysis:** Clinical needs and costs (i.e. acute care utilization for adults with SCD) **Intervention:** A multidisciplinary care team intervention, including monthly team meetings and development of individualized care plans (i.e. pain management plans) (distribution)	**Cost** A significant decrease in ED use of SCD patients, in individuals with a history of high ED use
58. Price et al. (2018)	**Australia** Quantitative study (Regression analysis of 383 patients)	Cardiovascular disease prevention Evaluating the adaptation of a phone-based cardiac coaching program for Greek and Italian populations	**Segmentation analysis:** Clinical and psychosocial needs **Intervention:** Adaptation of a coaching program according to cultural needs (e.g. education regarding diet differs between Greeks, Italians and English groups’ lifestyles) (distribution)	
59. Rose (2018)	**Australia** Quantitative study (Cross-sectional study of 250 patients from three radiotherapy departments)	Radiotherapy Assessing patients on their perceptions of individualized care provided by nurses	**Segmentation analysis:** Clinical, psychosocial and service needs **Intervention:** Patient characteristics, such as age, gender, and education may not predict how patients support tailored interventions Health messages achieving a level of personal relevance (e.g. technology awareness) to affect behavior change (distribution) **Conceptual basis:** Individualized care	
60. Rydback and Hyder (2018)	**Philippines** Qualitative study (Semi-structured interviews with 18 managers from health-care providers and supporting organizations)	Medical tourism A customized approach offering satisfactory health-care services to patients in unfamiliar settings	**Segmentation analysis:** Psychosocial and service needs (e.g. related to cultural values; visa extension; price transparency) **Intervention:** Concierge service that mitigates the negative impact of an unfamiliar context (e.g. transportation service (fabrication) Well-trained and multilingual staff answering patient preferences and addressing cultural values (distribution) **Conceptual basis:** MC in medical tourism	
61. Sawamura et al. (2013)	**Japan** Quantitative study (questionnaire survey)	Elderly Assessing the role of nursing home models in meeting resident preferences	**Segmentation analysis:** Clinical, psychosocial and service needs (e.g. preferences about wake-up, dressing assistance in the morning, meals, bathing, toilet assistance, and spare time) **Intervention:** Structural customization (unit-care model facilities in comparison to group-care model facilities and conventional facilities) **Conceptual basis:** Individualized care	**Patient experience** More choice in menus and activities programs for spare time in unit-care model and group care model facilities
62. Seeleman et al. (2015)	**The Netherlands** Qualitative study (Comparative case study analysis)	Transversal Assessing organizational responsiveness to diversity (i.e. diverse group needs that differ from the main population)	**Segmentation analysis:** Clinical, psychosocial and service needs **Intervention:** Organizational factors (i.e. leadership and performance measures at management level) A competent and diverse workforce (e.g. higher degree of linguistic and ethnic concordance between patients and staff) Responsiveness in care provision (e.g. specific needs for migrants) (fabrication, distribution) **Conceptual basis:** Organizational approach to diversity	
63. Seyyed Rasooli et al. (2013)	**Iran** Quantitative study (Interviews and questionnaires with 400 inpatients’ from internal and surgical units in teaching hospitals)	Hospitalized patients Assessing patient perceptions of nurses’ support for individualized care	**Segmentation analysis:** All types, but no clear definition **Intervention:** To adapt routine procedures in work organization, according to patient needs (in particular, for patient’s personal life outside hospital, i.e. occupation and social life) (distribution) **Conceptual basis**: Individualized care	
64. Suhonen et al. (2014)	**Finland** Quantitative study (Regression analysis of 874 nurses)	Elderly Investigating associations among nurses’ practices factors and individualized care for older people	**Segmentation:** Note clear definition, all needs **Intervention:** Nurses’ practices factors (ethical climate, professional practice environment and level of perceived individualized care) that improve individualized care (distribution)	
65. Swartz et al. (2017)	**Canada** Quantitative study (Regression analysis of 246 surgical or diagnostic procedures in 224 patients)	Children with autism spectrum disorder Assessing an individualized plan based on the provision of preoperative sedation, stratified by autism spectrum severity levels, before surgical procedures	**Segmentation analysis:** Clinical needs (i.e. level of autism spectrum disorder severity level) **Intervention:** A dedicated multidisciplinary and flexible perioperative program (e.g. cooperation of patients, adequate preparation) (distribution)	
66. Tuttle and Alzahrani (2019)	**International** Recommendations (literature review and author experience)	Thyroid cancer Implementing individualized care plans for patients with differentiated thyroid cancer	**Segmentation analysis:** Clinical needs (dynamic clinical risk assessment to guide all aspects of thyroid cancer management) **Intervention:** To tailor therapy and follow-up intensity to the estimated risks of recurrence and disease-specific mortality (distribution)	
67. Veterans Health Administration Transmittal Sheet (2018)	**USA** Recommendations (directive)	Lesbian, Gay, Bisexual, and Transgender (LGBT) Defining recommendations for customized care delivery to LGBT patients	**Segmentation analysis:** Clinical, psychosocial and service needs **Intervention:** Room assignments and specific access to facilities like restrooms (fabrication) Medical benefits package (e.g. hormonal therapy, pre- and post-operative evaluation) (assembly) LGBT coordinator (distribution)	
68. Vuik et al. (2016)	**United Kingdom** Qualitative study (Comparative analysis based on international case studies)	Transversal Proposing targeted population strategies in integrated care	**Segmentation analysis:** Clinical, psychosocial and service needs **Intervention:** Tailoring care models depending on a segmentation logic (e.g. data availability, cost of appropriate linked data set) (distribution) **Conceptual basis:** Population strategies in integrated care	
69. Williams et al. (2018)	**USA** Qualitative study (case study)	Genomic information Implementing population-based genomic medicine in an integrated learning healthcare system	**Segmentation analysis:** Clinical needs (i.e. genomic information) **Intervention:** Bioinformatics analysis of genomic information (fabrication) Multidisciplinary collaboration (primary care, specialist, clinical genomics team) (distribution) International standards that represent genomic data in EHR systems **Conceptual basis:** Precision medicine/ personalized medicine	
70. Wittink et al. (2018)	**USA** Quantitative study (Randomized trial in a healthcare delivery context with 60 patients)	Patients with multiple chronic conditions Assessing the impact of a customized intervention (a novel technology-based intervention called “Customized Care on stressor disclosure” - i.e. financial, safety, transportation stressors)	**Segmentation analysis:** Psychosocial needs (i.e. financial, safety and transportation stressors) **Intervention:** Web application to improve patient-general practitioner communications (fabrication)	**Patient experience** Improvements in the likelihood of stressor disclosure, without affecting visit length with the general practitioner

**Fig. 1:**
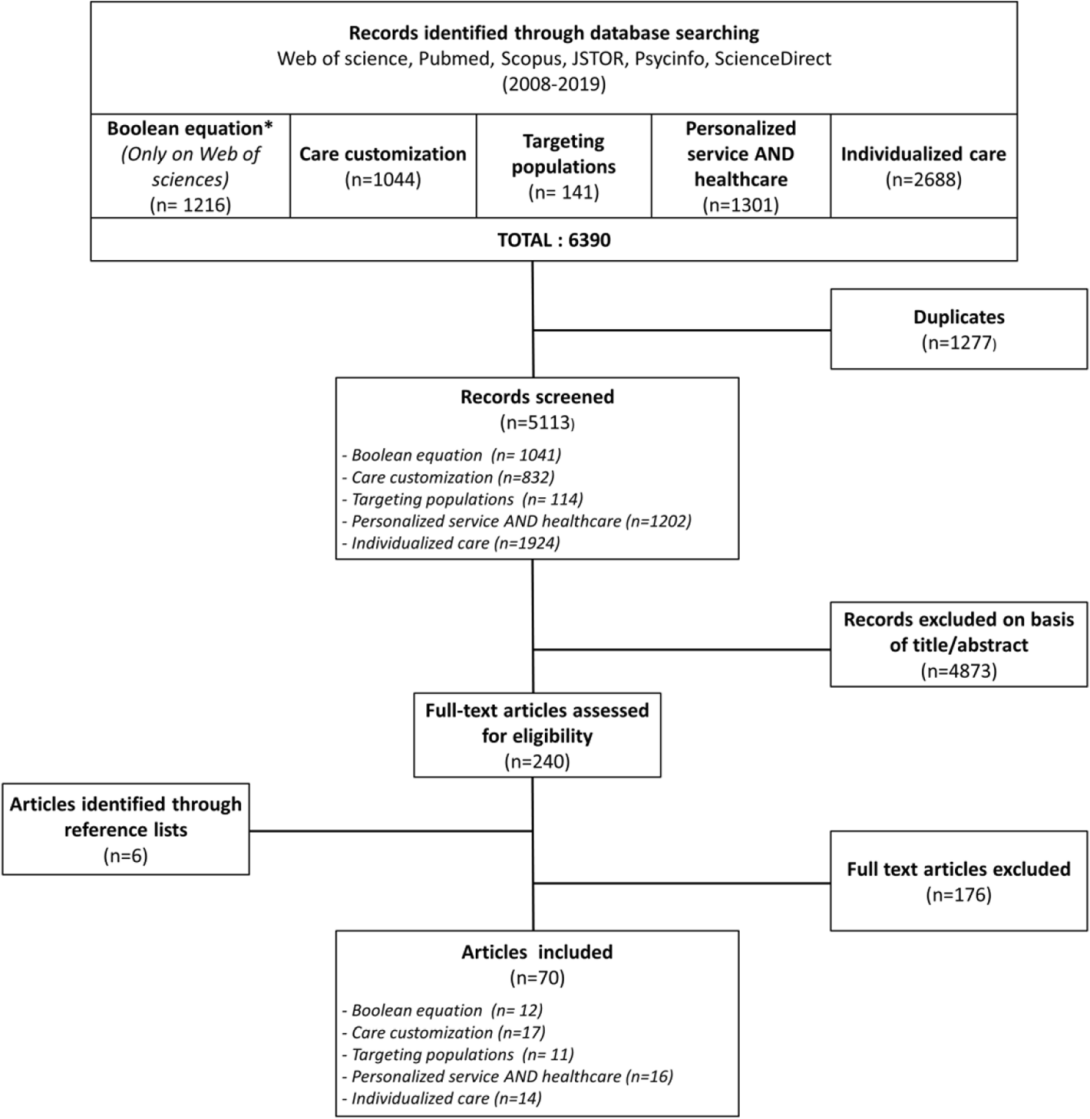
PRISMA flow diagram. * The Boolean search strategy introduced the search term “personalized medicine”. This search term refers to a very vast literature which does not allow it to be treated like the others. With the Boolean search strategy, it becomes possible to identify delivery issues related to “personalized medicine”

All the steps of the literature search, numbers of citations screened, duplicates removed and full-text documents assessed, are reported using a PRISMA flow diagram (Fig. 1).

## Results

The final selection included 70 articles. According to our framework, we have organized the results as follows: in a first part, we present the characteristics of the material; then, in a second part, we review the content of current efforts to implement care customization in delivery systems with reference to the concept of MC; finally, in a third part, we identify the potential conceptual basis of these efforts.

### Materials

Health topics varied from clinical conditions to socio-economic patient characteristics (i.e. gender characteristics; marginalizing conditions, immigrants). The most common health topics were cancer (*n* = 13) and the elderly (*n* = 9). Additionally, some articles (*n* = 8) addressed care customization strategies, using cost and resource allocation generated by patients (i.e. “Higher Utilizers” or “High needs High costs”). Lastly, some articles (*n* = 8) addressed care customization strategies in a general way, applicable to any type of patient (in this case, we use the world “transversal”).

Most articles (*n* = 42) were published between 2016 and 2019, with few published in the early years (i.e. five between 2008 and 2011, and 23 between 2012 and 2015). Approximately half the articles were based in the United States (*n* = 29). The remaining articles were outside the US: seven in Asia (China, India, Indonesia, Iran, Japan, Philippines, Taiwan), 20 in Europe (seven in the Netherlands, four in the United Kingdom, and one each in Denmark, Finland, France, Germany, Italy, Switzerland), and five across different countries representing international consortia.

### Care customization efforts

Two categories emerged when organizing the articles: those that applied care customization to an existing intervention (*n* = 28), and those that proposed the application of care customization to improve delivery systems (*n* = 48).

#### Segmentation analysis

We also categorized the articles according to the patient characteristics that support the segmentation analysis. Four categories emerged: (1) clinical needs (including genomic and molecular features); (2) psychosocial needs (i.e. needs due to financial and social status, psychological and personality traits, religious and cultural values; preferences related to behavioral attitudes in relation to health issues); (3) service needs (i.e. demands or preferences about aspects of life, apart from health issues); (4) costs (i.e. costs or resources allocated by the care process). These categories often overlapped, but for the purposes of this review, it was important to understand which patient characteristics were strongly informed by the segmentation analysis. Most articles reported on clinical needs (*n* = 51), but also psychosocial (*n* = 39) and service needs (*n* = 30) were covered. Clinical needs are often related to genomic characteristics, but they also cover new personalized plans, based on risk stratification (i.e. the decision to provide preoperative sedation is stratified by autism spectrum severity levels, before surgical procedures) [34]. Psychosocial needs included financial barriers (i.e. for: vision care appointments [35]; services related to social status [8, 36]; psychological characteristics (i.e. personality traits) [37, 38]; emotional attitudes [39]; and cultural values [40]. The service category emerged from analyses, while answers to patient demands were beyond what health care systems could usually deliver. Examples of service needs included home services for older persons, such as home energy assistance, legal services [41] or appointment scheduling [42].

Several types of patient characteristics could be identified during the segmentation analysis phase. Associations between different types of patient characteristics were distributed between as follow: homogeneously between 0, 1 and 2 associations and less for 3 associations (*n* = 5). The most usual situations were segmentation analyses based on clinical, psychosocial and service needs (*n* = 19), and clinical and psychosocial needs (*n* = 11). For example, Van der Laan et al. [43], in a study in Dutch older adults, defined five groups of patients based on a set of clinical and psychosocial need assessments. The assessment tool was proposed as a first triage step that offered more contextual information than purely disease-based information. The second most frequent segmentation analysis was based only on clinical needs (*n* = 12).

#### Customized interventions

Interventions were also categorized according to the three steps of any MC effort described in the theoretical part: (i) fabrication (i.e. any new care or service catering to the needs and demands of patients); (ii) assembly (i.e. any new structural combination of multiple standardized products and services, based on the needs and demands of patients) and (iii) distribution (i.e. any new coordination effort and integration of different products and services allowing timely delivery to patients). Examples of new care and services were various (*n* = 20): from concierge services offering transportation assistance for medical tourism [44], to new digital and artificial intelligence (AI) technologies that personalized physician choice for patients [45, 46], creating specific facilities depending on gender [47, 48], genomic tests [49–51] and biomedical informatics support [52]. Examples of assembly were less frequent (*n* = 7); one proposal aimed to combine care and services produced by two hospitals and an inpatient team in the same individualized care package, facilitating an inter-organizational infrastructure [53], nursing homes facilities [54], or accountable care organizations [55]. In contrast, distribution examples (*n* = 56) were mainly represented by coordination efforts in various clinical situations (i.e. during transitional care,) [56]. Distribution efforts also stressed communication efforts, in particular for personalizing preventive messages according to lifestyle [45, 57–61], or cancer survivorship [62]. Several studies also referred to the creation of multidisciplinary teams [63–65], including carers [66] and family members in specific conditions (i.e. to care for older persons with cognitive impairments) [67]. Stratification of interventions was also often reported, and was based on need requirements [68], and/or taking new needs and demands into account (i.e. behavioral and psychological characteristics) [69, 70]; personality traits [37, 71]; lifestyles for obesity prevention [61]; and cultural attitudes [62]. Organizational aspects like the organizational climate [72], the adaptation of routines [73], patient-nurse assortment [74], integration of new data and information in electronic health records [49, 75], were factors promoting customized interventions. Lastly, these distributional aspects were dependent on preferences and needs that could change over the course of care [76].

#### Impact evaluation

Several articles (*n* = 18) reported added-value evidence from customization. These were relatively equally distributed between patient experience (*n* = 7), cost (*n* = 6) and quality of care (*n* = 5), including quality of life [77]. However, none demonstrated customization, incorporating all three criteria.

### Theories

We observed that only 10% of articles explicitly applied MC theory for interventions (*n* = 6), and more generally that theoretical frameworks of any kind were often missing. Twenty-two different theories (i.e. inter-organizational collaboration or optimization of personalized assortment) informing segmentation and customized intervention were identified in 38 articles. Precision and personalized medicine (*n* = 5), individualized care (n = 6) and patient-centered care (n = 5) were the most represented theories.

## Discussion

In this scoping review, we identified 70 articles addressing how customization approaches are delivered in health care. Our results indicate the absence of a common strategy for delivering customized care based on a conceptual basis.

### The lack of a common strategy to deliver customized care

First, while the benefit of a customized delivery model is often advanced in recent years (the majority of papers have been published in the past 4 y), there is little conceptual basis that supports current efforts (the reference to a conceptual basis is only mentioned in about half of the studies). In this case, studies refer to a variety of models and theories (i.e., individualized care or consumer-centered care; patient-centered care or precision medicine; social marketing theories or models equity-oriented health care and “essential care”) [41, 77]. The theory of the “MC” is for its part little cited [24, 26, 78].

Second, the current efforts appear to be carried out in parallel, according to different logics of action. By logic of action, we refer to the definition of Strauss et al. [79] in which specific participants reflect common motivation for acting (i.e. categorizing patient characteristics and customized interventional responses) [79]. Depending on the focus given to one patient characteristic, or to the different association between them, four distinct logics of action can be defined: 1. Clinical; 2. Population health; 3. Patient-centered care and 4. Financial. Segmenting patients according to their clinical characteristics is “natural” in health care, and flows from the fact each patient is a single case. This clinical logic has recently been updated with the emergence of personalized and precision medicine, as highlighted by different articles [52, 80]. Beside this clinical logic, a second logic orients customization development to the psychosocial needs of the patient. In this instance, customization efforts have generally the goal to reduce inequalities to access and outcomes across populations. A good example of this comes from Ford-Gilboe et al. [42], who demonstrated that providing more equity-oriented health care improved health outcomes for people living in marginal conditions. The responsibility for structuring population health customization is most often borne by public health professionals, academics engaged with communities, local decision-makers, and social workers [81, 82]. The third logic, patient-centered care, describes how health care organizations respond to patient demands and preferences. In this context, care-giving is seen as a “service” on behalf of, and at the direction of patients. This logic is often referred to as “patient-centered care”, but in some cases is viewed through the notion of individualized care or marketing theories [44]. When patients are targeted by the costs their processes of care generate, they fall into a fourth financial logic. In this case, customization strategies have a common goal to target higher resource users, and to propose a customized intervention specifically to this subpopulation, applying it in various conditions. This financial logic generally refers to managers of health organizations and health systems who wish to rationalize costs. These four customization logics are not separate or independent, as evidenced by the combinations between clinical, psychosocial and service needs. The most common combinations observed in our review are segmentation analyzes associating clinical, psychosocial and service needs (*n* = 19), and clinical and psychosocial needs (*n* = 11). However, each logic reflects a potentially strong force that guides the division of work in health care. As such, they may create a set of parallel but unconnected approaches where participant groups develop a specific point of view of their organizational and professional goals, and similarly, the structure of their work follows this view, potentially undermining the promise of a common care customized delivery model. For example, the enthusiasm for personalized and precision medicine contributes to the spread of individually-focused clinical practices. This may, on its own, lead to quality improvements, and thereby advance the health of an individual population [83]. But this type of customization could be viewed by public health leaders as “premature” or inappropriately individualistic, thus missing out on the potential offered by broader applications of customization [84].

### Research and managerial implications

Given the nascent and heterogeneous state of the knowledge in this area, this is an important time to reflect on the definition and use of theoretical frameworks for building a theory-based customized care delivery model. We highlight three areas for researchers and managers involved interested in this field to move in this direction.

A first area of research could focus on the content of the segmentation analysis and the customized intervention steps in order to define a better conceptual basis. A first step consists in clarifying and increasing the characteristics of the patients taken into account during the segmentation analysis [67]. If we set aside the cost criterion which embodies another logic (i.e. the measurement of efficiency), many criteria are used to cover the needs, demands and preferences of patients. Beyond clarifying the concept of clinical and social needs [43], different articles stress the role of other patient characteristics: patient behaviors (i.e. the impact of negative personality traits in care delivery [37, 71], and preferences or demands expressed on subjects unrelated to health, but related to certain aspects of daily life, when encountering a health issue (i.e. lifestyle changes to prevent childhood obesity) [63]. They suggest that the usual characteristics of age, sex, clinical condition and education, may not predict how patients tolerate the adaptation of particular interventions [70], and call for opening up of patient characteristics used during segmentation analysis, and the type of data collected [69, 85]. In this consideration of a greater number of patient characteristics, modern profiling methods based on the processing of big data, may also have a role [46]. They can help develop forms of segmentation that identify the needs and demands of each patient [86]. This trend, if it is confirmed, could influence the conceptual basis of any care customization delivery model. More than a “MC” model (from mass to customization), it is a model of “singularity on a large scale” (from singularity to mass) that acknowledges the uniqueness of each patient by capturing a variety of needs and demands, which would serve as a conceptual basis.

Research could also help structure the content of a customized intervention. Several articles underlined the importance of coordination and communication efforts in customizing interventions, in particular by emphasizing the role of structural integration or developing multidisciplinary teams [63–65]. Other articles have stressed the importance of personalizing professional-patient relationships [87] while some others have stressed the key-role of modular packages [26]. The variety of actions listed during customization interventions calls for a more precise content, and how to apply them to the different stages of the patient pathway, within hospitals, but also during transitional care and primary care.

A second area of research relates to the added-value of a care customization delivery model. Our review reveals some positive impacts of care customization on quality, patient experience and costs. However, if we assume that more customization brings better outcomes and more satisfying experiences, the impact on costs requires more investigations. Investments for increased customization may bring additional costs at the different steps: investment in new methods of segmentation analysis (even if analytical algorithms can facilitate it) [88]; new services (i.e. new therapies, “concierge” systems, home services) [89]; or new forms of coordination between different health care professionals, managers, and social workers. Equally, several studies reported savings generated by customization, by reducing unnecessary hospitalization [56, 64], treatment costs [75], and duplication [90]. Other studies [8, 53] highlighted that an earlier and more focused identification of “complex” patients and/or high user can help health care organizations design more appropriate and efficient organizational responses. Chaudhuri and Lillrank [78] also argued that a customized strategy applied to high volumes of similar patients, and could be economic by implementing common standards of care. However, these data are sparse, and require more research of any care customization interventions to give evidence of their added-value, and in particular, of their economic viability [91].

A third last area of research could explore how to align the logics of action that underlie the efforts of care customization. While a single approach to customization of care is probably unrealistic, the compartmentalization of different logics may limit the impact of current customization efforts, resulting in additional costs. Researchers can help unravel the logics of action that support such developments, and find ways to facilitate their alignment, as in the case of customizing “high-need, high-cost patients” [8].

### Limitations

There are several limitations to this scoping exercise. Firstly, customization of care is rapidly growing and changing. There may be recent or more current efforts in developing interventions that have not yet appeared in the literature, or during our search, and as such, we may have inadvertently missed recently published articles. We excluded articles that addressed care customization, but not in routine health care delivery contexts (i.e. in clinical trials where they are not applied in real life contexts, fundamental research, or innovations at early development stages). As such, it is likely we underestimated the attributes of recent care customization strategies. The number of excluded clinical trials involving personalized medicine suggests these represent a dominant area of care customization in the mid-term.

Secondly, although we conducted an extensive literature review using a wide variety of terms capturing customization relevant articles, it is possible some articles may not have been identified. Our decision to include only studies that reported care customization may have excluded studies that addressed patient characteristics analysis in a customized effort, but were not labeled as such, or were not accurately represented in the abstract. To limit the impact of this selection process, we studied bibliographies of selected articles, and added six more relevant articles. We also selected only articles identifying customization strategies (i.e. segmentation analysis that led to a customized intervention) as the driver of this review, and we excluded segmentation initiatives that may have added insights (i.e. patient-profiling questionnaires and machine learning methods). In some cases, it was difficult to assess if segmentation analyses were accompanied by a customized intervention, or to circumscribe the notion of segmentation analysis itself, by comparison with stratification methods. To limit the incorrect exclusion of some articles, investigators discussed these cases. Last, we did not include “grey” literature; including newsletters, professional association or institutional news, and publicity publications. As care delivery systems and its analysis are not delineated research elements in medicine, this absence may have represented a bias that overlooked recently developed customized strategies, but not yet published. However, the analytical review of bibliographies in each selected articles did not uncover any more relevant information. Third, it is likely our selection process omitted specific research on one of the four customization approach steps, potentially missing important studies. For example, the definition of a “modular package” requires a better understanding of how the range of care and services could be combined into packages, and be pre-grouped as studies have shown [26, 27]. The same was also true for related issues such as care customization from regulatory and ethical perspectives (i.e. individual privacy, segmentation and discrimination of sub-populations based on ethnicity). These elements represent interesting research perspective for the future, and can help improve the conceptual of a customized care delivery model.

## Conclusions

This study is the first systematic review to examine current care customization as a new way of delivering health care and related services. The analysis shows that there is no theoretically-derived common strategy for delivering customized care. Consequently, we have identified three priority areas of research to advance in the development of a common customization delivery model: better define the content of the segmentation analysis and the intervention steps, demonstrate its added value, in particular its economic viability, and align the logics of action that underpin current efforts of customization. It would allow them to use customization to reduce costs and improve quality of care.

## Data Availability

The data sets analysed in this study are available from the corresponding author on request.

## Appendix 1

### Boolean equation - Web of sciences Search Strategy)

1. (care customization OR personalized medicineOR personalized service OR targeting populations OR individualized care)
2. AND (segmentation analysis OR profiling patients OR categorizing patients)
3. AND (patient needs OR patient characteristics OR patient preferences OR population characteristics OR patient demands)
4. limit 1 + 2 + 3 to English
5. limit 4 to Article
6. limit 5 to yr = 2008–2019

## Abbreviations

CRF: Cancer-related fatigue
ADHD: Children with attention-deficit/hyperactivity disorder
CGA: Comprehensive Geriatric Assessment
EHR: Electronic health record
ED: Emergency Department
HNHC: High needs high costs
ICPs: Individualized care plans
IT: Information technology
LGBT: Lesbian, Gay, Bisexual and Transgender
LDL: Low Density Lipoprotein
MC: Mass Customization
PCC: Patient-Centered Care
PRISMA: Preferred Reporting Items for Systematic Reviews and Meta-Analyses
SCD: Sickle cell disease

## References

[1] Peabody, F.W. The care of the patient. JAMA. 1927, doi: 10.1001/jama.252.6.813, 1984 10.1001/jama.252.6.813 6379210

[2] Hodson, R. Precision medicine. Nature. 2016, doi: 10.1038/537S49a. 10.1038/537S49a 27602738

[3] Hamburg, M.A., Collins, F.S. The path to personalized medicine. N. Engl. J. Med. 2010, doi; 10.1056/NEJMp1006304. 10.1056/NEJMp1006304 20551152

[4] Mead N, Bower P (2000). Patient-centeredness: a conceptual framework and review of the empirical literature. Soc Sci Med.

[5] Bardes, C.L. Defining 'patient-centered medicine'. N. Engl. J. Med., 2012, doi: 10.1056/NEJMp1200070. 10.1056/NEJMp1200070 22375968

[6] Coulter A, Entwistle VA, Eccles A, Ryan S, Shepperd S, Perera R (2015). Personalized care planning for adults with chronic or long-term health conditions. Cochrane Database Syst Rev.

[7] Suhonen, R., Välimäki, M., Leino-Kilpi, H. A review of outcomes of individualized nursing interventions on adult patients. J. Clin. Nurs., 2008, doi: 10.1111/j.1365-2702.2007.01979.x 10.1111/j.1365-2702.2007.01979.x 18321285

[8] Blumenthal, D., Anderson, G., Burke, S., Fulmer, T., Jha, A.K., Long, P. Tailoring complex-care management, coordination, and integration for high-need, high-cost patients: a vital direction for health and health care. NAM Perspect, 2016, doi: 10.31478/201609q

[9] McLaughlin CP, Kaluzny AD (2000). Building client centered systems of care: choosing a process direction for the next century. Health Care Manag Rev.

[10] Davis SM (1987). Future perfect. Reading.

[11] Pine, B.J. Mass customizing products and services. Plan. Rev., 1993, doi: 10.1108/eb054420

[12] Kotha, S. Mass customization: implementing the emerging paradigm for competitive advantage. Strateg. Manag. J., 1995, doi: 10.1002/smj.4250160916

[13] Da Silveira, G., Borenstein, D., Fogliatto, F.S. Mass customization: literature review and research directions. Int. J. Prod. Econ., 2001, doi: 10.1016/S0925-5273(00)00079-7

[14] Payne, A., Frow, P. A strategic framework for customer relationship management. J. Mark., 2005, doi: 10.2307/30166559

[15] Venter, P., Wright, A., Dibb, S. Performing market segmentation: a performative perspective. J. Mark. Manag., 2015, doi: 10.1080/0267257X.2014.980437

[16] Meyer, M.H., DeTore, A. Perspective: creating a platform-based approach for developing new services. J. Prod. Innov. Manag., 2001, doi: 10.1016/S0737-6782(01)00070-4

[17] Jitpaiboon, T., Dobrzykowski, D.D., Ragu-Nathan, T.S., Vonderembse, M.A.. Unpacking IT use and integration for mass customization: a service-dominant logic view. Int. J. Prod. Res. 2013, doi: 10.1080/00207543.2012.720727

[18] Zhang M, Zhao X, Qi Y (2014). The effects of supply chain integration on customer satisfaction and financial performance: an organizational learning perspective. Int.J Prod Econ.

[19] Gwinner, K.P., Bitner, M.J., Brown, S.W., Kumar, A. Service customization through employee adaptiveness. J. Serv. Res., 2005, doi: 10.1177/1094670505279699

[20] Schilling, M.A. Toward a general modular systems theory and its application to inter-firm product modularity. Acad. Manag. Rev., 2000, doi: 10.2307/259016

[21] Lampel J, Mintzberg H (1996). Customizing customization. Sloan Manag Rev.

[22] Mannion, R., Exworthy, M. (Re) making the Procrustean bed? Standardization and customization as competing logics in health care. Int. J. Heal. Policy Manag., 2017, doi: 10.15171/ijhpm.2017.35 10.15171/ijhpm.2017.35 PMC5458790 28812821

[23] Bohmer, R.M.J. Medicine’s service challenge: blending custom and standard care. Health care Manage Rev., 2005, doi: 10.1097/00004010-200510000-00006 10.1097/00004010-200510000-00006 16292009

[24] Minvielle E, Waelli M, Sicotte C, Kimberly JR (2014). Managing customization in health care: a framework derived from the services sector literature. Health Policy.

[25] Meyer MH, Jekowsky E, Crane FG (2007). Applying platform design to improve the integration of patient services across the continuum of care. Manag Serv Qual.

[26] De Blok C, Luijkx K, Meijboom B, Schols J (2010). Modular care and service packages for independently living elderly. Int J Oper Prod Manag.

[27] De Blok, C., Meijboom, B., Luijkx, K., Schols, J. The human dimension of modular care provision: Opportunities for personalization and customization. Int. J. Prod. Econ., 2013, doi: 10.1016/j.ijpe.2012.05.006

[28] Soffers R, Meijboom B, Van Zaanen J, Van Der Feltz-Cornelis C (2014). Modular health services: a single case study approach to the applicability of modularity to residential mental health care. BMC Health Serv Res.

[29] Silander, K., Torkki, P., Lillrank, P., Peltokorpi, A., Brax, S.A., Kaila, M. Modularizing specialized hospital services: constraining characteristics, enabling activities and outcomes. Int J Oper Prod Manag. 2017, do: 10.1108/IJOPM-06-2015-0365

[30] Tricco AC, Lillie E, Zarin W, O'Brien KK, Colquhoun H, Levac D, Moher D, Peters MDJ, Horsley T, Weeks L, Hempel S, Akl EA, Chang C, McGowan J, Stewart L, Hartling L, Aldcroft A, Wilson MG, Garritty C, Lewin S, Godfrey CM, Macdonald MT, Langlois EV, Soares-Weiser K, Moriarty J, Clifford T, Tunçalp Ö, Straus SE (2018). PRISMA extension for scoping reviews (PRISMA-ScR): checklist and explanation. Ann Intern Med.

[31] Arksey H, O'Malley L (2005). Scoping studies: towards a methodological framework. Int J Soc Res Methodol.

[32] Daudt HM, van Mossel C, Scott SJ (2013). Enhancing the scoping study methodology: a large, inter-professional team’s experience with Arksey and O’Malley’s framework. BMC Med Res Methodol.

[33] Hart C (2009). Doing a literature review: releasing the research imagination.

[34] Swartz JS, Amos KE, Brindas M, Girling LG, Graham MR (2017). Benefits of an individualized perioperative plan for children with autism spectrum disorder. Pediatr Anesth.

[35] Aggarwal S, Ju D, Allen AM, Rose LA, Gill KP, Shen SA, Temko JE, Chang I, Faraj J, Brabender DE, de Cortina SH, Marik-Reis O, Mehta MC (2018). Regional differences in vision health: findings from Mwanza, Tanzania. Int Health.

[36] Seeleman C, Essink-Bot ML, Stronks K, Ingleby D (2015). How should health service organizations respond to diversity? A content analysis of six approaches. BMC Health Serv Res.

[37] Friedman B, Veazie PJ, Chapman BP, Manning WG, Duberstein PR (2013). Is personality associated with health care use by older adults?. Milbank Q.

[38] Lattie EG, Asvat Y, Shivpuri S, Gerhart J, O’Mahony S, Duberstein P, Hoerger M (2016). Associations between personality and end-of-life care preferences among men with prostate cancer: a clustering approach. J Pain Symptom Manag.

[39] Hagan TL, Arida JA, Hughes SH, Donovan HS (2017). Creating individualized symptom management goals and strategies for cancer-related fatigue for patients with recurrent ovarian cancer. Cancer Nurs.

[40] Price P, Tacey M, Koufariotis V, Stramandinoli D, Vincent R, Grigg L, Zentner D (2018). A contemporary phone-based cardiac coaching program: evolution and cross cultural utility. Hear Lung Circ.

[41] Golden RL, Emery-Tiburcio EE, Post S, Ewald B, Newman M (2019). Connecting social, clinical, and home care services for persons with serious illness in the community. J Am Geriatr Soc.

[42] Ford-Gilboe M, Wathen CN, Varcoe C, Herbert C (2018). How equity-oriented health care affects health: key mechanisms and implications for primary health care practice and policy. Mibank Q.

[43] Van der Laan MRE, van Offenbeek MAG, Broekhuis H, Slaets JPJ (2014). A person-centred segmentation study in elderly care: towards efficient demand-driven care. Soc Sci Med.

[44] Rydback M, Hyder AS (2018). Customization in medical tourism in the Philippines. Int J Pharm Healthc Mark.

[45] Larsen LB, Sondergaard J, Thomsen JL, Halling A (2019). Step-wise approach to prevention of chronic diseases in the Danish primary care sector with the use of a personal digital health profile and targeted follow-up - an assessment of attendance. BMC Public Health.

[46] Pan X, Song J, Zhang F. Dynamic recommendation of physician assortment with patient preference learning. IEEE Trans Autom Sci Eng. 2019. 10.1109/TASE.2018.2839651.

[47] Lin HC. A study on women’s perceptions regarding the requirements and satisfaction of a hospital environment. GMBHS. 2011. 10.1016/j.gmbhs.2011.09.001.

[48] VHA Directive 1341, Providing health care for transgender and intersex veterans. 2018. https://www.albuquerque.va.gov/docs/ProvidingHealthcareforTransgenderandIntersexVeterans.pdf

[49] Jing X, Kay S, Marley T, Hardiker NR, Cimino JJ (2012). Incorporating personalized gene sequence variants, molecular genetics knowledge, and health knowledge into an EHR prototype based on the continuity of care record standard. J Biomed Inform.

[50] Hassett MJ, Silver SM, Hughes ME, Blayney DW, Edge SB, Herman JG, Hudis CA, Marcom PK, Pettinga JE, Share D, Theriault R (2012). Adoption of gene expression profile testing and association with use of chemotherapy among women with breast cancer. J Clin Oncol.

[51] Manegold C (2014). Treatment algorithm in 2014 for advanced non-small cell lung cancer: therapy selection by tumor histology and molecular biology. Adv Med Sci.

[52] Barlow-Stewart K (2017). Personalized medicine: more than just personal. AQ Aust Q.

[53] Hardin L, Kilian A, Spykerman K (2017). Competing health care systems and complex patients: an inter-professional collaboration to improve outcomes and reduce health care costs. J Interprof Educ Pract.

[54] Sawamura K, Nakashima T, Nakanishi M (2013). Provision of individualized care and built environment of nursing homes in Japan. Arch Gerontol Geriatr.

[55] O’Malley AS, Rich EC, Sarwar R, Schultz, et al. How accountable care organizations use population segmentation to care for high-need, high-cost patients. Issue Brief (Commonw. Fund). 2019:1–17. 30645057

[56] Jenq GY, Doyle MM, Belton BM, Herrin J, Horwitz LI (2016). Quasi-experimental evaluation of the effectiveness of a large-scale readmission reduction program. JAMA Intern Med.

[57] Brandzel SD, Bowles EJA, Wieneke A, Bradford SC (2017). Cancer screening reminders: addressing the spectrum of patient preferences. Perm J.

[58] Geller BM, Shelly JM, Dorwaldt AL, Howe (2008). Increasing patient/physician communications about colorectal cancer screening in rural primary care practices. Med Care.

[59] Gesser-Edelsburg A, Shalayeva S (2017). Internet as a source of long-term and real-time professional, psychological, and nutritional treatment: a qualitative case study among former Israeli Soviet Union immigrants. J Med Int Res.

[60] Gordon TF, Bass SB, Ruzek SB, Wolak C (2014). Developing a typology of African Americans with limited literacy based on preventive health practice orientation: implications for colorectal cancer screening strategies. J Health Commun.

[61] Kolodinsky J, Reynolds T (2009). Segmentation of overweight Americans and opportunities for social marketing. Int J Behav Nutr Phys Act.

[62] Blanch-Hartigan D, Viswanath K (2015). Socioeconomic and sociodemographic predictors of cancer-related information sources used by cancer survivors. J Health Commun.

[63] Avis JL, Ambler KA, Jetha MM, Boateng H (2013). Modest treatment effects and high program attrition: The impact of interdisciplinary, individualized care for managing pediatric obesity. Pediatr Child Health.

[64] Mercer T, Bae J, Kipnes J, Velazquez M (2015). The highest utilizers of care: individualized care plans to coordinate care, improve health care service utilization, and reduce costs at an academic tertiary care center. J Hosp Med.

[65] von Dadelszen P, Magee LA, Payne BA, Dunsmuir DT, Drebit S (2015). Moving beyond silos: how do we provide distributed personalized medicine to pregnant women everywhere at scale? Insights from PRE-EMPT. Int J Gynecol Obstet.

[66] Braaf SC, Lennox A, Nunn A, Gabbe BJ (2017). Experiences of hospital readmission and receiving formal carer services following spinal cord injury: a qualitative study to identify needs. Disabil Rehabil.

[67] Petry H, Ernst J, Steinbrüchel-Boesch C, Altherr J (2019). The acute care experience of older persons with cognitive impairment and their families: a qualitative study. Int J Nurs Stud.

[68] Pilotto A, Cella A, Pilotto A, Daragjati J (2017). Three decades of comprehensive geriatric assessment: evidence coming from different health care settings and specific clinical conditions. J Am Med Dir Assoc.

[69] McConnell H, White R, Maher J (2017). Categorizing cancers to enable tailored care planning through a secondary analysis of cancer registration data in the UK. BMJ Open.

[70] Rose PM (2018). Patients’ characteristics informing practice: improving individualized nursing care in the radiation oncology setting. Support Care Cancer.

[71] Cotrell V, Carder PC (2010). Health-related needs assessment of older residents in subsidized housing. Cityscape..

[72] Papastavrou E, Acaroglu R, Sendir M, Berg A (2015). The relationship between individualized care and the practice environment: an international study. Int J Nurs Stud.

[73] Seyyed Rasooli A, Zamanzadeh V, Rahmani A, Shahbazpoor M (2013). Patients’ point of view about nurses’ support of individualized nursing care in training hospitals affiliated with Tabriz University of Medical Sciences. J Caring Sci.

[74] Dewi WN, Evans D, Bradley H, Ullrich S (2014). Person-centred care in the Indonesian health-care system. Int J Nurs Pract.

[75] Williams MS, Buchanan AH, Davis FD, Faucett WA (2018). Patient-centered precision health in a learning health care system: Geisinger’s genomic medicine experience. Health Aff (Millwood).

[76] dos Reis S, Park A, Ng X, Frosch E, Reeves G (2017). Caregiver treatment preferences for children with a new versus existing attention-deficit/hyperactivity disorder diagnosis. J Child Adolesc Psychopharmacol.

[77] McCabe MP, Beattie E, Karantzas G, Mellor D (2019). Consumer directed care in residential aged care: an evaluation of a staff training program. Aging Ment Health.

[78] Chaudhuri A, Lillrank P. Mass personalization in health care & colon; insights and future research directions. J Adv Manag Res. 2013. 10.1108/JAMR-05-2013-0033.

[79] Strauss A, Fagerhaugh S, Suczek B, Wiener C (1982). The work of hospitalized patients. Soc Sci Med.

[80] Topol EJ (2019). Deep medicine : how artificial intelligence can make health care human again.

[81] Kertesz SG, Holt CL, Steward JL, Jones RN, Roth DL, Stringfellow E, Gordon AJ, Kim TW, Austin EL, Henry SR, Johnson NK, Granstaff US, O’Connell JJ, Golden JF, Young AS, Davis LL, Pollio DE (2013). Comparing homeless persons’ care experiences in tailored versus nontailored primary care programs. Am J Public Health.

[82] Volpp KG, Krumholz HM, Asch DA (2018). Mass customization for population health. JAMA Cardiol.

[83] Bayer R, Galea S (2015). Public health in the precision-medicine era. N Engl J Med.

[84] Horton R (2018). Offline: in defence of precision public health. Lancet.

[85] Vuik SI, Mayer EK, Darzi A (2016). Patient segmentation analysis offers significant benefits for integrated care and support. Health Aff.

[86] Dekkers T, Hertroijs DFL. Tailored health care: two perspectives on the development and use of patient profiles. Adv Ther. 2018. 10.1007/s12325-018-0765-2. 10.1007/s12325-018-0765-2 PMC6133138 30105659

[87] Hunter PV, Hadjistavropoulos T, Thorpe L, Lix LM (2016). The influence of individual and organizational factors on person-centred dementia care. Aging Ment Heal.

[88] Basu A, Carlson JJ, Veenstra DL (2015). A framework for prioritizing research investments in precision medicine. Med Decis Mak.

[89] Dorfman EH, Brown Trinidad S, Morales CT, Howlett K (2015). Pharmacogenomics in diverse practice settings: implementation beyond major metropolitan areas. Pharmacogenomics.

[90] Züger G, Honegger F (2014). Essential requirements for the parameterization of food waste in hospitals. Int J Facil Manag.

[91] Padula WV, Millis MA, Worku AD, Pronovost PJ, Bridges JFP, Meltzer DO (2017). Individualized cost-effectiveness analysis of patient-centered care: a case series of hospitalized patient preferences departing from practice-based guidelines. J Med Econ.

